# Maze design: size and number of choices impact fish performance in cognitive assays

**DOI:** 10.1111/jfb.15493

**Published:** 2023-07-14

**Authors:** Nick A. R. Jones, Daphne Cortese, Amelia Munson, Helen C. Spence‐Jones, Zoe Storm, Shaun S. Killen, Ruth Bethel, Amy E. Deacon, Mike M. Webster, Libor Závorka

**Affiliations:** ^1^ Department of Animal Physiology University of Bayreuth Bayreuth Germany; ^2^ Centre for Social Learning and Cognitive Evolution, School of Biology, University of St Andrews St Andrews UK; ^3^ School of Biodiversity, One Health and Veterinary Medicine, University of Glasgow Glasgow UK; ^4^ Alfred‐Wegener‐Institut Helmholtz‐Zentrum für Polar‐ und Meeresforschung, Wadden Sea Station Sylt List Germany; ^5^ Department of Life Sciences The University of the West Indies St Augustine Trinidad and Tobago; ^6^ WasserCluster Lunz – Biologische Station, Inter‐university Centre for Aquatic Ecosystem Research Lunz am See Austria; ^7^ Danube University Krems Krems Austria

**Keywords:** choice arena, cognition, fish, minnows, plus‐maze, spatial cognition, sticklebacks, T‐maze

## Abstract

Although studies on fish cognition are increasing, consideration of how methodological details influence the ability to detect and measure performance is lagging. Here, in two separate experiments the authors compared latency to leave the start position, latency to make a decision, levels of participation and success rates (whether fish entered the rewarded chamber as first choice) across different physical designs. Experiments compared fish performance across (a) two sizes of T‐mazes, large and standard, and a plus‐maze, and (b) open choice arenas with either two or four doors. Fish in T‐mazes with longer arms took longer to leave the start chamber and were less likely to participate in a trial than fish in T‐mazes with shorter arms. The number of options, or complexity, in a maze significantly impacted success but did not necessarily impact behavioural measures, and did not impact the number of fish that reached a chamber. Fish in the plus‐maze had similar latencies to leave the start box and time to reach any chamber as fish in the same‐sized T‐maze but exhibited lower overall success. Similarly, in an open choice arena, increasing the number of options – doors to potential reward chambers − resulted in lower probability of success. There was an influence of reward position in the choice arena, with rewarded chambers closest to the sides of the arena resulting in lower latencies to enter and higher probability of decision success. Together the results allow the authors to offer practical suggestions towards optimal maze design for studies of fish cognition.

## 1 INTRODUCTION

Cognition – encompassing the mechanisms by which animals acquire, process, store and act on information – is a crucial mechanism for coping with the surrounding environment, including rapid adaptation to environmental changes (Shettleworth, 2009), and is thus of interest to multiple research fields. Nonetheless, with increasing research effort has come a growing awareness of the challenges inherent in measuring cognitive performance, particularly, methodological issues that can affect comparability between studies and assays, and the difficulty in accounting for inter‐ and intra‐individual variation (Boogert et al., 2018; Chittka et al., 2009; Griffin et al., 2015; Rowe & Healy, 2014; Thornton & Lukas, 2012). A broad range of methodological factors can impact the data acquisition and repeatability of behavioural measurement across studies, which are likely to, in turn, impact performance in cognitive assays. For example, latencies to leave the “start” chamber can depend on the size of the start box (Näslund, Bererhi, & Johnsson, 2015), whereas the swimming speed and time spent in shelter can depend on enclosure size (Polverino et al., 2016). “Within‐experiment” methodological factors that can impact cognitive research in fishes have been highlighted as a key area to address (Salena et al., 2021).

Any experimental study aiming to explore cognition must balance the capture of meaningful measures of cognitive performance against the practical and logistical constraints of experimental work (*e.g*., time budgets or space constraints). Experimental methodology may introduce bias; in some tests, individual variation in performance may reflect differences in motivation (Thornton & Lukas, 2012; van Horik and Madden, 2016), exploratory tendencies (Guillette et al., 2009) or stress (Koolhaas et al., 1999) rather than cognitive differences, generating variation in performance which does not reflect variation in the cognitive trait under study. Similarly, tests which represent an extreme of cognitive (or physiological) ability in a species (*i.e*., too “easy” or too “challenging”) will fail to capture variation present within the population as all individuals are likely to perform similarly: for example, common octopus (*Octopus vulgaris*) fail in discrimination trials based on colour, not because they are incapable of discrimination between stimuli in general, but because they are colourblind (Messenger, 1977) (*i.e*., the test is too challenging). Notably, visual discrimination assays with two cues, although routinely used, are considered to be insufficiently challenging to detect variation, with the majority of individuals able to solve problems “at a glance” (Chittka et al., 2009; Wang et al., 2015). Participation rates are one particular area of consideration − physical design can potentially affect whether individuals ever leave a start area and engage with a trial. If some individuals never engage or participate, it is not possible to capture the cognitive variation they reflect in a population. Inter‐individual variation in cognition, and the association between cognition and behavioural traits (a.k.a. personality), is in itself an area of active research (Dougherty & Guillette, 2018; Lucon‐Xiccato & Bisazza, 2017b; Mendelson et al., 2016; Sih & Del Giudice, 2012) where it is of particular importance to capture meaningful variation between individuals (Boogert et al., 2018; Thornton & Lukas, 2012). There are general measures to reduce these biases, such as repeated testing across multiple assays (Boogert et al., 2018), increased reporting of potential sampling bias (Webster & Rutz, 2020), refined statistical analyses and interpretation (Farrar et al., 2023) and improvements in automated tracking of aquatic animals (Dutta et al., 2023). Nonetheless, other methodological details, such as the physical designs of assays, are potentially important considerations.

Despite the variety of designs used in cognitive studies, there are startlingly few published studies that have explicitly tested the effects of physical designs commonly used and the potential impact of modifications to them. Among the wide variety of experimental assays used in cognitive research, many include some form of spatial navigation. Assays utilizing door or chamber choices based on navigation and/or cue presence are common across a wide range of disciplines: T‐mazes (or similar “Y” or “plus” mazes) are widely used (Benvenutti et al., 2021; Cleal et al., 2021), alongside larger and less‐restrictive variants, such as water mazes for studies on rodents (Vorhees & Williams, 2014) or radial‐arm mazes (Paul et al., 2009). Nonetheless, outside of work on a select few model species (notably rats, Fernandes & File, 1996; Friske & Gammie, 2005; Reichelt et al., 2021; and mice, Hodges, 1996; O'Leary & Brown, 2012; O'Leary & Brown, 2013), there are few published studies that explore the effects of maze design on performance captured by these “typical” assays. A large and growing number of fish species are commonly used in laboratory studies of cognition across a wide variety of fields (Braasch et al., 2015; Brown et al., 2011; Bshary et al., 2001; Schartl, 2014; Utne‐Palm & Smith, 2020; Vila Pouca & Brown, 2017). Mazes and spatial tests of cognition are also commonly used in studies with fish (Brown et al., 2011; Bshary & Brown, 2014; French, 1942; Lucon‐Xiccato & Dadda, 2014). A recent review brought attention to the need for greater consideration of the physical design of maze‐type assays in zebrafish, *Danio rerio* (Hamilton 1822), (Benvenutti et al., 2021), and many of these concerns are applicable to the cognitive testing of fish in general. The review found that other factors that need to be considered are the differences in the dimensions, shapes and material of mazes used in different studies. A better understanding of how such discrepancies in physical design influence the measurement of fish cognition and behaviour is urgently needed to advance the study of cognition across fish species.

Here, the authors explore the impacts of maze design on performance in two tests of spatial cognition carried out on two different fish species. They used two fish species across two labs to explore the general effects of physical design of mazes on fish performance. They initially intended to cover additional species and labs, but due to COVID lab closures, they were unable to fulfil these plans. The study was not intended to explicitly compare results found in the literature, but to explore effects of physical design on commonly used cognition assays. In one experiment they tested the effect of the arm length in a T‐maze (short arm T‐maze *vs*. long‐arm T‐maze), and the effect of an additional arm (standard two‐choice T‐maze *vs*. three‐choice maze, a.k.a. a “plus” maze) using the threespine stickleback *Gasterosteus aculeatus* (L. 1758). In the second one, they tested the effect of maze complexity, in terms of number of choice chambers (two or four) in an arena maze, on the performance of common (or European) minnows *Phoxinus phoxinus* (L. 1758). In both cases, individual fish were repeatedly tested once a day for 20 days in a spatial learning task, where they had to locate a food reward (bloodworm).

## 2 METHODS

### 2.1 T‐mazes with threespine sticklebacks

#### 2.1.1 Subjects and husbandry

A total of 90 fish of undetermined sex were tested across three maze designs. Wild threespine sticklebacks, *G. aculeatus*, were collected from the Kinnessburn stream in St Andrews, UK (56.3362 N, −2.7916 E) using passive funnel‐traps set overnight to ensure a representative sample of behavioural types as passive traps can be biased when set out for short durations due to the level of sociality of an individual (Kressler et al., 2021). Fish were collected and tested in three successive batches such that 30 fish were collected and tested at a time. Fish were selected with body lengths between 4.5 and 5.0 cm and were not in breeding condition. Each fish was housed individually in a 45 l aquarium for 2 weeks to allow for acclimatization to the laboratory conditions, and during testing. Each housing aquarium was aerated with an air stone and contained the same physical enrichment (Supporting Information Figure S1 shows an image of the housing tanks). As per the DETAILS enrichment reporting framework (Jones et al., 2021) each tank was furnished with gravel (mixed brown colour, grain size 3–5 mm, 0.5 cm depth covering 100% of the bottom of the tank), one artificial plant (8 cm tall, light green leaves, 2 cm maximum leaf breadth), one PVC tube (grey, 1 cm diameter, 5 cm long) and an overhead shelter in the form of a black opaque plastic sheet covering one‐third of the surface area of the tank (the preferred form of shelter for sticklebacks; Jones et al., 2019). Housing and experimental water was maintained at 10.0°C, with a 12L:12D photoperiod.

Fish were fed with small (10 mm) defrosted bloodworms (Chironomid larvae) at the end of each day, *i.e*., once any experimental trials were completed. Due to limited housing conditions, fish were collected and tested in three successive batches of 30 fish each. In each batch fish were pseudo‐randomly allocated to and tested in one of the maze designs to ensure 10 fish in each maze per batch. After testing was complete, batches of fish were housed in group tanks until the three batches of fish had all been tested. They were then released back into the wild at the point of capture.

#### 2.1.2 Experimental set‐up

**FIGURE 1:**
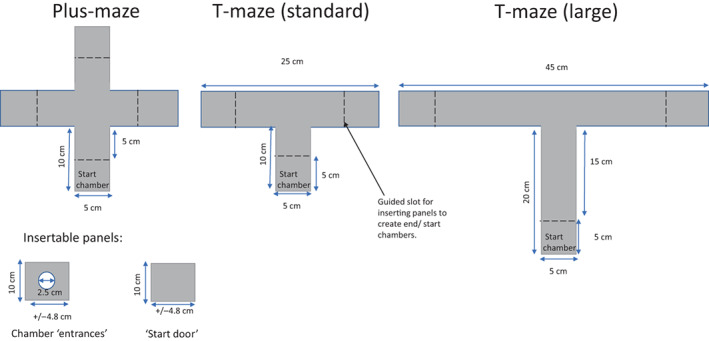
Dimensions of each maze, used to test impact of maze design on sticklebacks, the plus‐maze, standard T‐maze and large T‐maze.

Three types of T‐mazes were used, each constructed from opaque grey plastic (see Figure 1): a “standard” two‐choice maze, a “large” two‐choice maze and a three‐choice maze (a.k.a. a “plus” maze). The size of the large and plus mazes was made relative to the “standard size,” which was based on the maze sizes used in previous studies of cognition in three‐spine stickleback, specifically Mamuneas et al. (2015) and Odling‐Smee and Braithwaite (2003a). The authors doubled the standard arm length for the larger maze to ensure a difference in distance that is detectable by the fish, with fish having to swim three body lengths to reach the end of an arm compared to two. Each of the test arms in the maze had a removable panel insert with a 2.5 cm diameter entrance cut through the middle, to allow entrance while preventing the fish from seeing the contents of the chamber from outside. The starting chamber had a solid panel insert which was removed to begin the trial. One test chamber contained a single bloodworm as a food reward (the “correct” chamber), which was randomly assigned prior to the experiment. The maze walls were all 10 cm high, and during trials the water depth was 8.5 cm.

#### 2.1.3 Procedure

After each fish had been assigned to a maze design treatment, they were consistently tested in that maze. Similarly, the reward position for each fish was fixed so that fish could be expected to learn the position of the food reward. Reward condition was balanced so that an equal number of fish were rewarded in either the left, right or (for the plus‐maze) top end chambers throughout. In both the standard and large mazes, 15 fish were assigned to each reward position side (*i.e*., left and right chambers), whereas in the plus‐maze 11 fish were assigned to the left, 10 to the right and 9 to the top end chambers. During trials fish were run three at a time, one in each maze type. For each maze, the bloodworm was placed at the bottom of the allocated reward chamber before each trial, and any floating pieces of bloodworm were removed. Two drops of water that had been used to defrost and soak with bloodworms were added to the non‐rewarded chambers of each maze to minimize olfactory cues as a means by which the fish could locate the reward. The fish were then placed in their respective “start chamber” and left for 1 min to acclimatize [this acclimation period was based on a pilot study and previous studies (Jones et al., 2019)]. The authors also provided an overhead shelter in the start chamber which can aid handling recovery, where the start chamber “door” partition was then removed by hand and positioned on top of the start chamber such that it formed an overhead shelter for the fish. The fish were then left for a 12‐min trial period. Fish behaviour was recorded using a video camera (USB 5‐megapixel) mounted 1.5 m above the mazes. Fish that did not find or consume the bloodworm were not given a reward. After the trials, the fish were then returned to their housing tanks and the mazes rinsed out and refilled with new aerated water from a reservoir tank at the same temperature (10.0°C). The water changes were performed to eliminate social cues and scents that could influence fish in subsequent trials. Each fish was tested in a single trial per day for 4 weeks, with a 2‐day break over the weekends, such that each fish was tested in a total of 20 trials in their respective maze type and reward position.

#### 2.1.4 Measures

Using the videos, the authors scored the following measures for each trial for each fish: (a) the latency to leave the start box, measured as the number of seconds taken until the fish's whole body had emerged from the shelter above the start chamber. In some instances fish rapidly left the start chamber as the door was opened in a “flight” response and then maintained a frozen position, a common response to being startled (Kalueff et al., 2013; Näslund, Lindström, et al., 2015). In these instances, the authors scored latency as time to begin swimming freely; (b) the choice latency, measured as the number of seconds to enter any chamber (head up until first dorsal spine); (c) decision success (binomial) – whether the fish entered the rewarded chamber first or not; and (d) participation (binomial) − whether the fish entered any chamber. Non‐performers were given the maximum trial latency and a zero for success.

### 2.2 Arena mazes with minnows

#### 2.2.1 Subjects and husbandry

Common minnows (*P. phoxinus*) of undetermined sex were collected from the River Kelvin (Glasgow, 55° 52′ 42″ N 004° 17′ 03″ W) in September 2021 using dip‐nets and minnow traps and immediately brought to the lab at the University of Glasgow. Fish were kept in laboratory conditions (14°C, and 12 L:12 D photoperiod). Gravel was used as substrate enrichment (“natural” mixed colour pea gravel, grain size 4–16 mm) covering 100% of the bottom of the tanks. Fish were initially housed in 42 L stock tanks (*n* = 50 fish per tank); after 2 weeks, all fish were anesthetized in a solution of benzocaine and water (5 ml of benzocaine solution per litre of water) and tagged with a visible implant elastomer (Northwest Marine Technology, Inc) with a two‐colour code using a combination of pink, yellow, red, blue, green, orange, violet, white. Two weeks post‐tagging, 39 fish were moved to two smaller aerated tanks (27 l) containing gravel and three plastic plants (9 cm tall with green leaves, leave size: 35 mm length and 3 mm width). Supporting Information Figure S2 shows an image of the minnow housing tanks. Experimental trials were conducted in two rounds; one (*n* = 18 fish) began a week after transfer to post‐tagging tanks (*i.e*., 21 days post‐tagging), and the other (*n* = 21 fish) began 20 days after transfer (*i.e*., 41 days post‐tagging). Minnows were retained in the laboratory for future use. Fish were fed bloodworm *ad libitum* during pre‐experiment housing and at the end of the experimental days, after all trials were complete.

#### 2.2.2 Experimental set‐up

**FIGURE 2:**
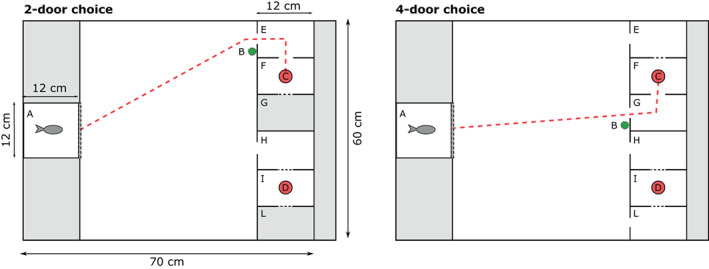
Diagram of the arena set‐up for testing the effect of the number of doors on minnow performance. Each arena included a starting chamber (A) with a door that was lifted after the acclimation period and a small plastic plant used as a landmark (B) to indicate the reward chamber (F) (dashed red line shows direct path to reward) containing the accessible food reward (C). An additional Petri dish with bloodworm (D) was placed in the closed reward chamber (I) to control for visual and olfactory cues. In the two‐door maze, there were two open compartments (E and H), but the interior reward chamber was blocked with a mesh door in one and open in the other. Similarly, in the four‐door maze, there were four open compartments (E, G, H, L) but three contained blocked entrances to the reward chamber, and only one was open.

An 80 × 60 cm arena (based on Závorka et al., 2020) was used for trials, with either two or four initial door chambers that opened to two potential reward chambers (Figure 2). Fish were initially moved to a 12 × 12 cm starting chamber before being released into the main maze arena. Both solid and mesh removable panel inserts (*i.e*., doors; 12 × 4.5 cm) were used to manipulate visibility of reward and number of choices available. In the less‐complicated maze, there was always exactly one closed door between the two open doors (*i.e*., there was always an open door on the edge and the centre). Each “reward” chamber contained one bloodworm anchored in Vaseline on an inverted Petri dish (35 mm diameter) and were accessible only *via* choice chambers; one was the true reward chamber, whereas the other was a decoy chamber blocked by mesh doors to provide a control for visual and olfactory cues of the bloodworm. They also used a landmark cue in the form of a small (3 cm tall) green plastic plant attached to the right side of the “correct” choice chamber in each trial.

The arena was lined with white corrugated plastic, and all sides were painted white with aquarium safe paint (coloured waterproof sealant; brand: Liquid Rubber) to maximize contrast with fish for video analysis. A black curtain surrounded the maze area to minimize disturbances. Water temperature was maintained at 14°C throughout trials. Between each trial, water was removed and replaced with fresh 14°C water from a temperature‐controlled reservoir tank.

#### 2.2.3 Procedure

A total of 39 fish were used in experimental trials, of which 33 completed all 20 days of training (*n* = 19 for two‐choice maze, *n* = 16 for four‐door maze). Fish were removed if they exhibited breeding colours or aggression towards tank mates. Each fish was randomly assigned to a maze design at the start of the experiment and tested in the same maze design throughout. The correct chamber and positions of the doors were also kept constant for each fish. Three mazes were used, allowing three fish to be tested at a time. For each maze, the bloodworms were placed in the reward and decoy chambers prior to the experiment, as well as the plant cue for the “correct” door. The fish was then placed in the “start chamber” and left for 5 min to acclimatize. The start chamber “door” partition was then removed remotely using a pulley system, and the fish were left for 15 min to explore the maze. Fish behaviour was recorded using a video camera (GoPro 4 or 7) mounted *c*. 1 m above the mazes. The fish were then returned to their housing tanks. Every fish was tested in one trial per day for 20 consecutive days.

#### 2.2.4 Measures

Using manual scoring, the authors scored video recordings of each trial for each fish: [Corrections added on 13 October 2023, after first online publication: This sentence has been corrected in this version.] (a) the choice latency, measured as the number of seconds to enter any chamber; (b) decision success (binomial) − whether the fish entered the rewarded chamber as a first choice; and (c) participation (binomial) − whether the fish entered any chamber. Non‐performers were labelled as NA for latency scores and a zero for success.

### 2.3 Statistical analyses

All analyses were conducted using R base package (R Core Team, 2022). The lme4 package (Bates et al., 2015) was used to fit the multi‐level mixed models (GLMM), and *post hoc* Tukey pair‐wise comparisons (where appropriate) were conducted using the emmeans package (Lenth et al., 2018).

#### 2.3.1 T‐mazes with sticklebacks

##### Maze comparison

The effect of maze design on behavioural performance was explored using latency to leave the start chamber, latency to enter any chamber (choice latency) and proportion of fish that completed a trial (*i.e*., participation) as response variables. Maze design, the three types of maze tested, was set as the explanatory variable. Trial number was incorporated as a random factor in all models, and fish ID was included as a random factor for all models of success.

To explore effects of maze on cognitive performance, the authors fitted trial success (whether the rewarded chamber was chosen first) against maze design. Trial number and fish ID were used as random factors.

Note that the authors excluded fish that were rewarded in the top arm of the plus‐maze for the overall maze comparison. They noticed running trials there that fish seemed to enter the “top” chamber more quickly, and apparently more frequently than other chambers. They analysed the effect of reward position within the plus‐maze separately (see below).

##### Plus‐maze reward position

For the plus‐maze the authors checked whether the latency to leave the start chamber, and success rates, differed with reward position across the three end chambers.

#### 2.3.2 Arena mazes with minnows

To explore the effect of maze design on common minnows' behavioural performance the latency to enter any chamber (choice latency) and proportion of fish that completed a trial (*i.e*., participation) were used as response variables. Maze design (categorical variable with two levels: two *vs*. four chambers) and position of the reward, either centre (chambers G and H) or external (chambers E and L, closest to the sides of the arena), were used as explanatory variables. Trial number and fish ID were used as random factors. Model residual distributions were checked by visual inspection. We excluded trials where latency to leave the start chamber was less than 2 seconds. Cognitive performance was analysed in the same manner as sticklebacks above.

### 2.4 Ethical notes

The experiments described here were conducted in accordance with the requirements of ASAB/ABS Guidelines for the care and use of animals and to comply with the ARRIVE guidelines for using animals in research (du Sert et al., 2020). Each experiment was conducted after approval from the respective ethics governing body of the institution involved: the Animal Welfare and Ethics Committee of the University of St Andrews and the University of Glasgow Animal Welfare and Ethical Review Board (project licence PB948DAA0).

## 3 RESULTS

### 3.1 Experiment 1: T‐style mazes with sticklebacks

#### 3.1.1 Behavioural performance

**FIGURE 3:**
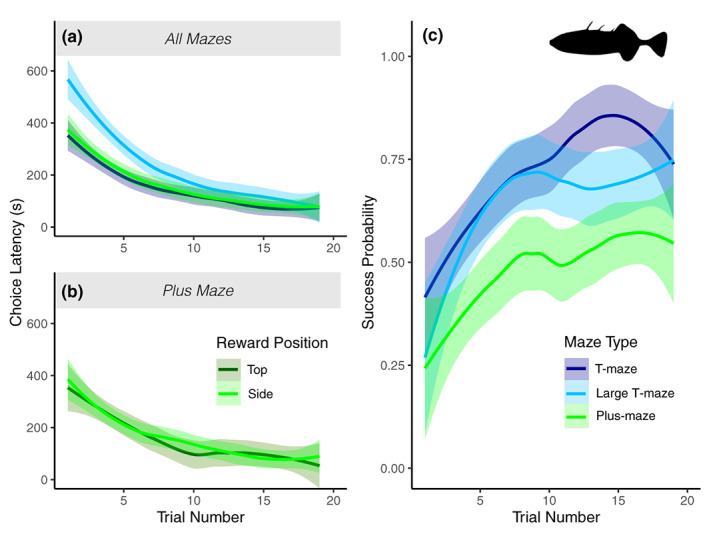
Threespine stickleback performance is affected by maze design: (a) latency to enter any chamber across maze types, (b) latency across reward position in the plus‐maze only and (c) the probability of a successful trial across different maze types. Lines represent the loess smoothed conditional mean with 95% c.i. (shaded area) for each maze tested. *N* = 27 in standard T‐maze, *N* = 28 in large T‐maze and *N* = 29 in the plus‐maze.

The time that fish took to leave the start chamber was significantly affected by the maze type across the three mazes tested (LRT: $\chi_{2}^{2}$ = 32.9, *P* < 0.001). *Post hoc* contrasts revealed that fish in the larger T‐maze (with longer arms) had significantly greater latencies to leave the start box than fish in mazes with shorter arms (both T‐ and plus‐mazes); standard T‐maze (emmean contrast: −0.316, *P*< 0.001); and plus‐mazes (emmean contrast: −0.482, *P* < 0.001). The level of participation (*i.e*., whether a fish entered at least once chamber during the trial, regardless of if it was the rewarded chamber) was also significantly affected by maze (LRT: $\chi_{2}^{2}$_2_ = 18.89, *P* < 0.001); fewer sticklebacks entered a chamber in the long‐arm maze when compared to the smaller T‐maze (emmean contrast: +0.636, *P* = 0.006), or plus‐maze (emmean contrast: +0.213, *P* = 0.002). There were, however, no significant differences in the proportion of fish that entered a chamber between the standard T‐maze and the plus‐maze (*P* = 0.3859). For those trials where a fish did enter a chamber, the time taken to reach any chamber (rewarded or not) was likewise affected by maze type (LRT: $\chi_{2}^{2}$ = 30.8, *P* < 0.001; Figure 3a) with fish taking more time in the larger T‐maze.

#### 3.1.2 Cognitive performance

The number of successful trials, where a fish enters the “correct” rewarded chamber before any other chamber, was also impacted by maze type (LRT: $\chi_{2}^{2}$ = 16.23, *P* < 0.001; Figure 3c). *Post hoc* comparisons show that fish in the plus‐maze had significantly lower success than fish in both the standard T‐maze (emmean contrast: +1.627, *P* < 0.001) and the large T‐maze (emmean contrast +1.153, *P* = 0.013). Overall frequency of success across trials did not differ significantly between the standard and large versions of the T‐maze (*P* = 0.431). Note that success was not dictated by chance: success was at or below chance for all mazes in the initial trial, but higher than expected by chance in the final trial (see Supporting Information Table S1).

#### 3.1.3 Effect of reward position in the plus‐maze

The position of the reward did not affect the latency to leave the start position in the plus chamber (LRT: $\chi_{2}^{2}$ = 0.041, *P* < 0.980; Figure 3b). Nonetheless, reward position was a significant factor that impacted success for fish in the plus‐maze (LRT: $\chi_{2}^{2}$ = 16.68, *P* < 0.001; Supporting Information Figure S3). Fish that were trained with the food reward in the chamber opposite to their start position (top chamber) were significantly more likely to be successful than fish that were trained with the reward in either the left or right chambers (emmean contrast −0.760, *P* = 0.005 and emmean contrast – 0.987, *P <* 0.001 respectively).

### 3.2 Experiment 2: arena mazes with minnows

#### 3.2.1 Behavioural performance

**FIGURE 4:**
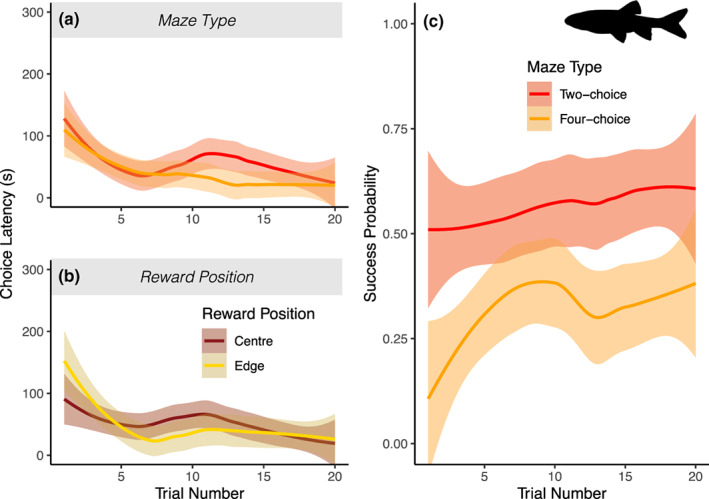
Minnows are affected by number of doors in an arena test: (a) latency to enter any door across the two arena configurations, (b) effect of reward position on latency to enter any door, (c) the probability of a successful trial differs across the different arenas. Lines represent the loess smoothed conditional mean with 95% c.i. (shaded area) for each maze tested. *N* = 16 in four‐door arena, and *N* = 19 in the two‐door arena. [Corrections added on 17 October 2023, after first online publication: Figure 4 has been updated in this version.]

Choice latency by minnows was not affected by design aspects of the arena (either number of choices or position of reward). The time that minnows took to enter any chamber was not significantly affected by the number of chambers in the arena (LRT: $\chi_{1}^{2}$ = 1.587, *P* = 0.207; Figure 4a). Likewise, there was no significant effect of reward position (‘edge’ or ‘centre’) on choice latency (LRT: $\chi_{1}^{2}$ = 0.437, *P* = 0.508; Figure 4b). [Corrections added on 13 October 2023, after first online publication. This paragraph has been updated for accuracy in this version.] The participation rates also did not differ between the two arenas; the proportion of fish that entered any door during the test was not significantly different between four‐ and two‐door mazes (LRT: $\chi_{2}^{2}$ = 0.32382, *P* = 0.569).

#### 3.2.2 Cognitive performance

As with sticklebacks, the probability of “success” − a fish entering the rewarded chamber before any other chamber − was also impacted by maze type (LRT: $\chi_{2}^{2}$ = 16.23, *P* < 0.001; Figure 4c). Nonetheless, there was a strong interaction effect with reward position, RewardPosition:Numberof Doors (LRT: $\chi_{1}^{2}$ = 15.647, *P* < 0.001): fish in the four‐door arena with the reward in one of the centre chambers had significantly lower success than the fish in the other treatment and reward position combinations (*P* < 0.001).

## 4 DISCUSSION

The results show that multiple attributes of mazes can have important effects on fish participation and performance in cognitive trials which researchers must consider when refining experimental protocols. In both minnows and sticklebacks, increasing complexity (number of available choices) had a negative effect on overall success rates (proportion of correct choices; Figure 3c and 4c [Corrections added on 13 October 2023, after first online publication: Figure 3 citations have been corrected in this version.]). Increasing maze size, by contrast, did not affect success rate in sticklebacks –but did increase latency to leave the start chamber (Figure 3b) and decreased participation rates overall (*i.e*., a higher proportion of fish tested never entered a test chamber during the trial period). Together, these results highlight important factors for researchers to consider when designing and interpreting experimental studies. In particular, a smaller maze may allow more efficient use of experimental time as it reduces required trial times without impacting observed learning performance, while altering maze complexity may allow for calibration of success rates to best capture more meaningful measures of cognitive performance. Such design refinements may also offer an alternative to employing negative cues – such as electric shocks – as a method of improving participation and performance of fish subjects in behavioural trials (Ngoc Hieu et al., 2020).

Both minnows and sticklebacks had a lower overall success rate in more complex mazes, with more choice options. This is in accordance with expectations (Chittka et al., 2009) and comparison of two‐choice options in other fields (Bruzzone & Corley, 2011; Dougherty, 2020). This effect may explain some apparent contradictions within the literature on speed/accuracy trade‐offs related to behavioural phenotypes impacting success in cognition assays; studies which do not find such a trade‐off are often conducted with two‐choice assays (Kareklas et al., 2017; Lucon‐Xiccato & Bisazza, 2016; Mamuneas et al., 2015; Trompf & Brown, 2014), whereas studies with three or more choices do find evidence for such trade‐offs across a range of taxa (Chittka et al., 2003; Cussen & Mench, 2014; DePasquale et al., 2016; Jones et al., 2020; Moiron et al., 2016). More complex mazes, which represent a “challenging middle” for a species, may allow us to measure increased inter‐individual variation in success, thereby revealing correlations with other aspects of the phenotype which would be hidden in two‐choice mazes where the majority of individuals show similar (high) performance. The plus‐maze and the four‐door arenas in these experiments both showed lower initial and final success rates than their lower complexity counterparts. This suggests that they may offer a simple solution to increasing the challenge of a design. Nonetheless, running experiments in these mazes for longer and setting learning criteria will be required to test this speculative point more fully. It would be interesting to see if the observed difference in the success rate between the T‐ and plus‐mazes was driven purely by chance initially, *i.e*., the success rate impacted by the number of options, and whether given enough time success rates reach that of the mazes with two options – or whether some additional factors make the plus‐maze design more difficult to solve compared to the T‐maze. As a final note, an intriguing pattern seen in both the stickleback and minnows was that in the more complex maze tasks (three‐ and four‐choice mazes), there was a “dip” in average success rates shortly after the learning maximum had been reached (Figures 3c and 4c). This may be indicative of the fish testing their learned assumptions once they had established them, or an increase in sampling of alternate potential food locations (Krebs et al., 1978), as shown for other fish species (Warburton, 1990).

Although maze complexity had significant effects on success rate in both species tested, it did not appear to impact the numbers of fish that could be counted as performers. Participation rates (the probability of a fish entering a test chamber during the trial) were unaffected by maze complexity in both species. The time taken to complete the trial – either in terms of latency to leave the start chamber or time to make a decision in similar‐sized mazes − was also not impacted by an additional end chamber in sticklebacks. [Corrections added on 13 October 2023, after first online publication: This sentence has been updated in this version.]. These results support the idea that altering maze complexity to adjust success rates need not come at the cost of increased experimental time or resources, in terms of number of individuals required to reach a baseline participation rate. Nonetheless, increased maze complexity may introduce greater potential for reward‐positional effects; in both the plus‐maze (sticklebacks) and the four‐door arena maze (minnows), fish showed bias for entering particular chambers (the “top” chamber in a plus‐maze and the “edge” chambers in an arena), which may be due to thigmotaxis (in open arenas) or an aversion to changing direction (in plus‐mazes). In the case of the plus‐maze with an additional arm, there was a significantly increased probability of a correct decision when the reward was placed in the top, “favoured” position. This bias may be overcome experimentally (*e.g*., by only rewarding the side arms in a plus‐maze) or should be investigated and/or controlled for statistically in subsequent analyses. It is worth noting that this study's results comparing T‐ and plus‐mazes of equal sizes show that the top position acts as a distractor position and lowers success even when the rewards are only in one of the two side chambers.

Maze dimensions, in terms of the length of the arms of the T‐maze, reduced participation rates and impacted latency. Fish tested in the longer‐arm T‐maze were less likely to choose a chamber and showed increased latency to leave the start chamber than those in the shorter‐arm T‐maze. Nonetheless, it did not impact overall success rates: there was no difference in probability of correct choice for sticklebacks between large‐ and small‐arm T‐mazes. This suggests that there are practical benefits to considering the size and dimensions of mazes. For example, in this study the T‐maze with shorter arms may allow researchers to run quicker learning trials and record more fish, as even “slow‐style” fish are more likely to leave the start box and participate in trials within a given trial constraint. It is also worth noting that the latency to leave the start chamber equalized by the end of the trials, and the observed effect of increasing arm length in the T‐maze, is likely driven by interactions between maze and behavioural traits such as boldness, and shelter‐seeking, and thigmotaxis (“wall‐hugging”) (Bailey et al., 2021; Bensky et al., 2017; Doria et al., 2019; Kallai et al., 2007; Lucon‐Xiccato et al., 2019; Lucon‐Xiccato & Bisazza, 2017a) or associated individual differences in activity levels.

This study shows that even relatively small differences in design can have a significant impact on fish performance in widely used assays of spatial cognition. Nonetheless, it is important to recognize that there can be other factors at play (Odling‐Smee & Braithwaite, 2003b). For example, the social and physical environment can play a role in behavioural and cognitive performance. The social environment can have dramatic influence on cognition, both directly and indirectly *via* behaviour. For example, latency to emerge from a start chamber can depend on previous social context (Jolles et al., 2016) and, threespine sticklebacks kept in solitary conditions display changes in gene expression of specific brain regions after just 1 week (Greenwood & Peichel, 2015). Similarly, age and sex of fish can impact performance – again sticklebacks provide clear examples whereby males and females exhibit differences in cognitive performance (Keagy et al., 2019). Finally, fish that have been housed with levels of environmental complexity or predator regimes can exhibit differences in cognitive performance (DePasquale et al., 2021; Odling‐Smee & Braithwaite, 2003b; Pereira et al., 2020; Salvanes et al., 2013; Spence et al., 2011), and behavioural responses such as measures of behavioural lateralization (Sundin et al., 2023). Broad generalizations from the results of this study should be made with care, as the authors studied animals from a single population in each experiment. It is possible that captive bred fish may exhibit fewer marked differences in latency to emerge from the start box, for example.

## 5 CONCLUSION

Mazes and choice arenas are widely used in assays of cognition, which are critical to develop our understanding of how animals learn about and adapt to changes in their environment. The authors hope that this study can provide empirical guidance on decisions regarding design choice and help researchers develop designs to best capture variation without reducing the number of participants. The results reveal that aspects of maze design – complexity and length of arms in a T‐maze – can be used to adjust expected performance in behavioural and cognitive trials for fish. Specifically, maze complexity appears to affect overall success rates without impacting participation or speed of completion, whereas maze size affects the latter two without impacting the former. The authors also illustrate that increasing maze complexity may introduce bias in terms of reward‐position effects, which should be taken into consideration in experimental design and analysis. Overall, the results demonstrate empirically the importance of methodological considerations in studies aiming to capture cognitive and behavioural performance in fish, particularly those aiming to capture inter‐individual variation in learning rates and discrimination success.

## AUTHOR CONTRIBUTIONS

Nick A. R. Jones, Daphne Cortese, Amelia Munson and Libor Závorka conceived of the study; Nick A. R. Jones, Daphne Cortese, Amelia Munson, Ruth Bethel, Amy E. Deacon, and Libor Závorka planned experiments; Shaun S. Killen, Mike M. Webster, Amy E. Deacon and Libor Závorka received funding, provided resources and supervision; Nick A. R. Jones, Daphne Cortese, Amelia Munson, Zoe Storm, Ruth Bethel ran the experiments and/or scored the videos; Nick A. R. Jones, Helen C. Spence‐Jones, Daphne Cortese and Amelia Munson conducted the statistical analyses; all authors contributed to drafting the manuscript.

## Supporting information


**DATA S1.** Supporting information
