# Supplementary material for: Maze design: size and number of choices impact fish performance in cognitive assays
Source: J Fish Biol. 2023 Jul 14;103(5):974–84. doi: 10.1111/jfb.15493 (PMC10952265; doi:10.1111/jfb.15493)
Supplement: Supplementary file 1 — DATA S1. Supporting information [file JFB-103-974-s001.docx]

**Table S.1** Success per maze compared to that expected by chance based on the number of end chamber options.

| Maze type | First trial |  |  | Last trial |  |  | Chance |
| --- | --- | --- | --- | --- | --- | --- | --- |
|  | Mean | sd |  | Mean | sd |  |  |
| Standard T-maze | 0.45 | 0.51 |  | 0.708 | 0.464 |  | 0.5 |
| Large T-maze | 0.231 | 0.439 |  | 0.75 | 0.442 |  | 0.5 |
| Plus-maze | 0.3 | 0.483 |  | 0.474 | 0.513 |  | 0.3 |


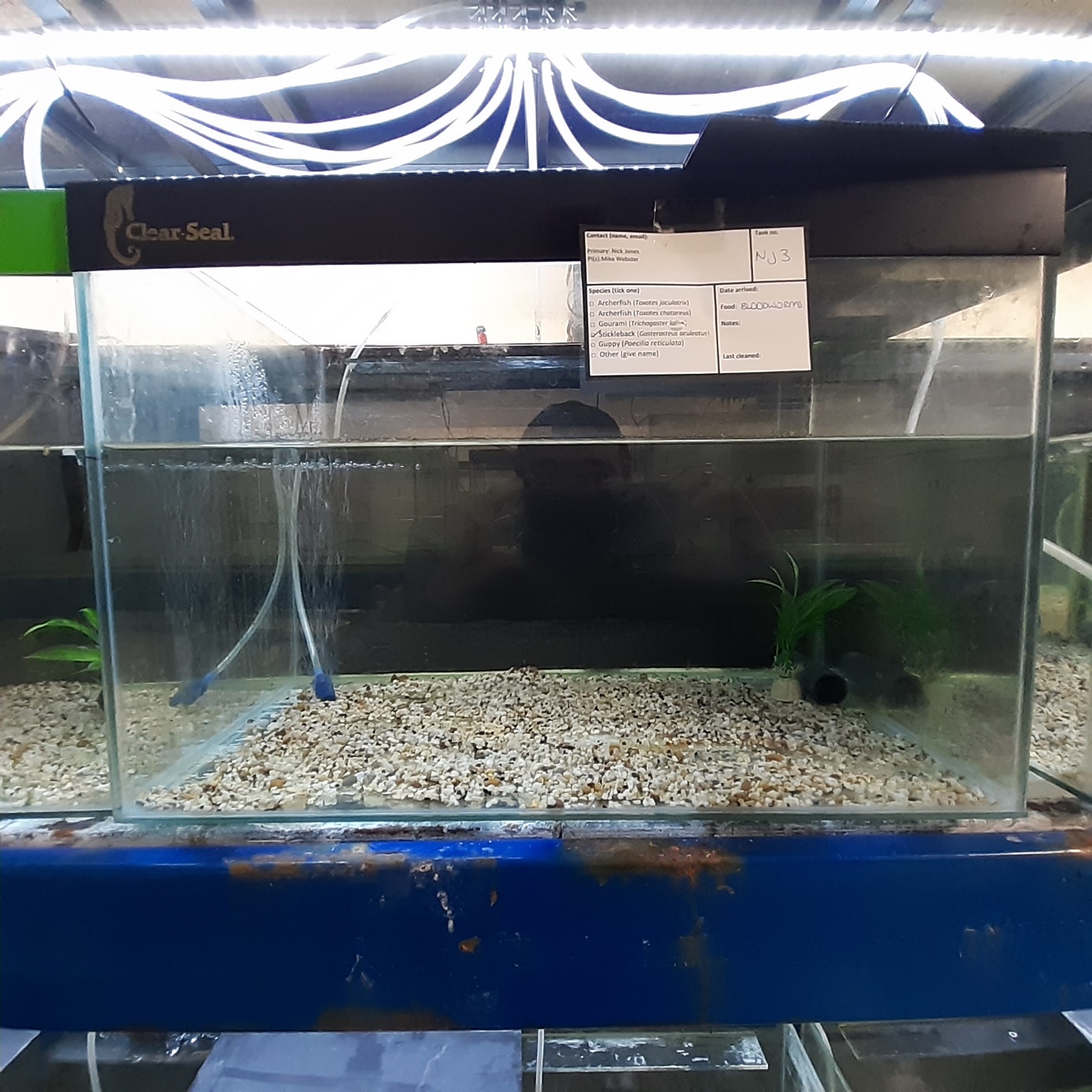


**Figure S.1 Housing conditions of solitary housed three-spined sticklebacks for the duration of the experiment.**


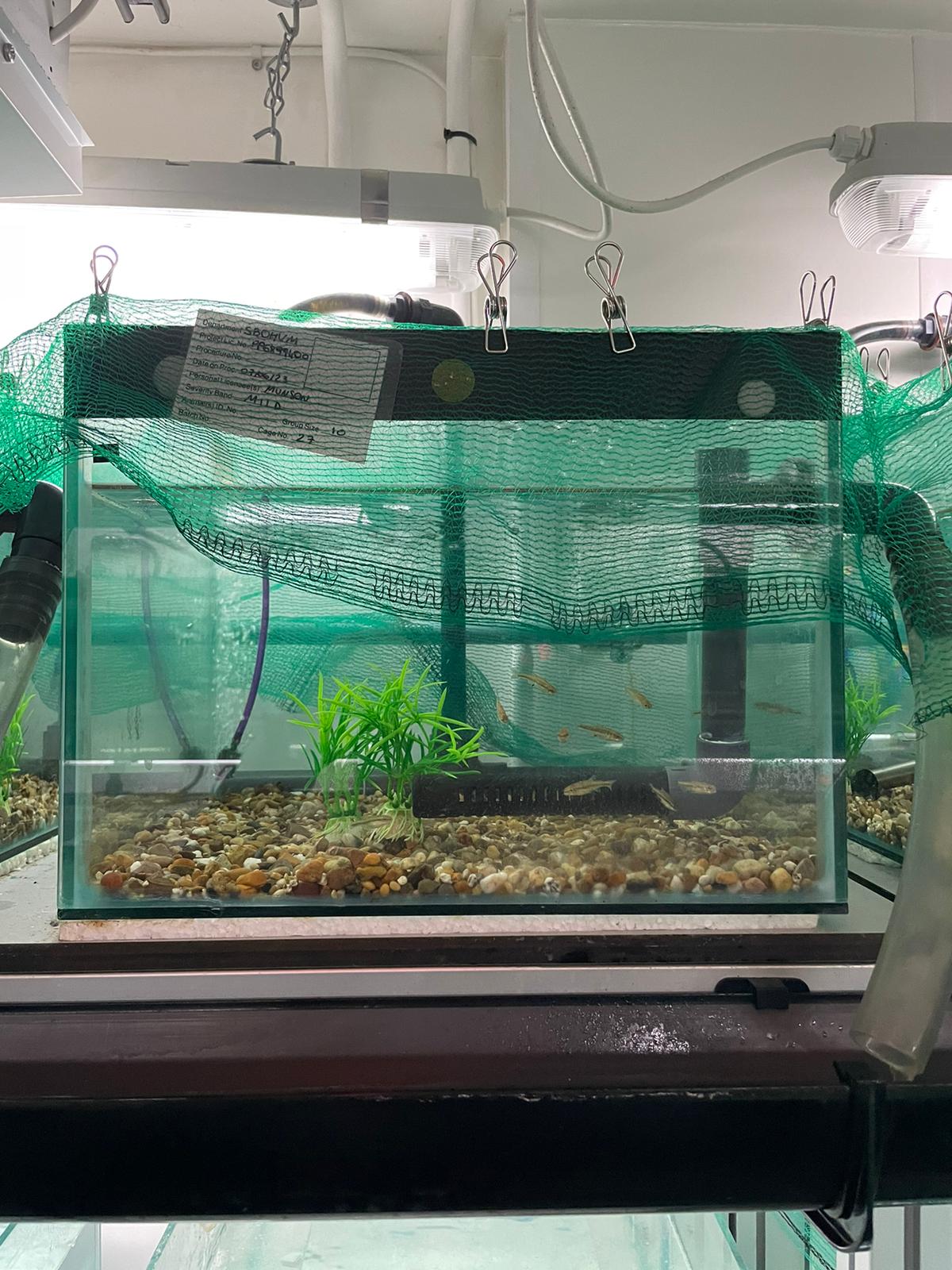


**Figure S.2 Housing conditions of European minnows for the duration of the experimental trials.**


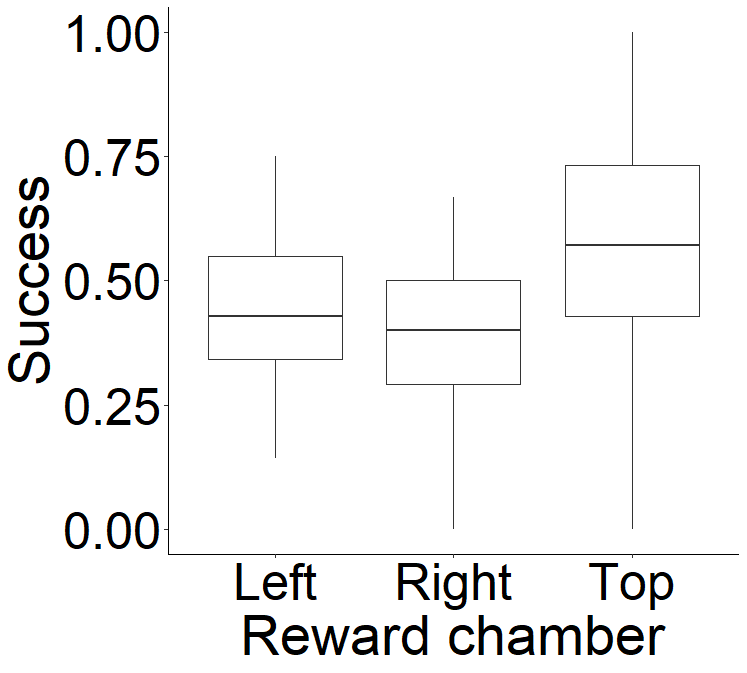


**Figure S.3 Mean success per trail across the three reward positions in the plus maze configuration.** Box plots show the median and 25th and 75th percentiles, the whiskers extend to the data point that is no more than 1.5 times the length of the box as per the Tukey method (R package ggplot2) and the circles are outliers.
